# Role of nursing and midwifery in mainstreaming genomics in Australia: mixed-methods study exploring scope of practice and strategies for implementation

**DOI:** 10.3389/fgene.2025.1717520

**Published:** 2026-01-05

**Authors:** Kim E. Alexander, Morgan J. Farley, Brighid Scanlon, Jed Duff

**Affiliations:** 1 School of Nursing, Faculty of Health, Queensland University of Technology, Brisbane, QLD, Australia; 2 Cancer and Palliative Care Outcomes Centre, Queensland University of Technology, Brisbane, QLD, Australia; 3 Cancer Care Services, Metro North Hospital and Health Service, Herston, QLD, Australia; 4 Faculty of Health, University of Technology Sydney, Sydney, NSW, Australia; 5 Centre for Clinical Research, University of Queensland, Herston, QLD, Australia; 6 Centre for Healthcare Transformation, Queensland University of Technology, Brisbane, QLD, Australia; 7 Royal Brisbane and Women’s Hospital, Queensland Health, Brisbane, QLD, Australia

**Keywords:** genomic-informed care, genomics, mainstreaming, nursing and midwifery, service delivery

## Abstract

**Background:**

The integration of genomics into Australian health services offers significant benefits for the diagnosis and delivery of targeted treatments, but its success relies on a workforce equipped to deliver genomic-informed care. Nurses and midwives, Australia’s largest healthcare workforce, have the potential to play a key role in the integration of genomic care into mainstream services by enabling access to the diverse and geographically spread Australian population. To achieve this, it is imperative to clearly define their roles in genomic practice and identify specific educational and resource needs.

**Methods:**

A two-part, mixed-method study was conducted encompassing a state-wide survey and a range of semi-structured interviews. The state-wide survey (n = 81), aimed to establish agreement on the key domains of genomic-related care practice for nurses and midwives. The semi-structured interviews with key practice change stakeholders (n = 32) sought to identify the barriers and facilitators to implementing the genomic practice domains. Descriptive statistics were generated to summarise the quantitative findings. The qualitative data were analysed using content analysis, with the findings organised according to the Consolidated Framework for Implementation Research.

**Results:**

There was general agreement (>85%) that nurses and midwives should be involved, to varying extents, in 18 of the 31 domains of genomic-related care. The domains with the highest overall agreement included being able to identify clinical indicators of genetic susceptibility, ability to take a family history and have a general understanding of genomic information. The integration of genomics into nursing and midwifery faces several barriers, including unclear roles and responsibilities, system and organisational challenges, and a lack of tailored education. Key facilitators identified were dedicated nursing or midwifery roles, tailored, co-designed education, and collaboration with key stakeholders.

**Conclusion:**

Our findings highlight the need for clearly defined roles and scope of practice for nurses and midwives, supported by tailored, co-designed workforce development programs and implementation processes. Such approaches are essential to meet the varied needs and competencies of nurses and midwives and to fully enable the benefits of accessible genomic care.

## Graphical Abstract

**Figure:**
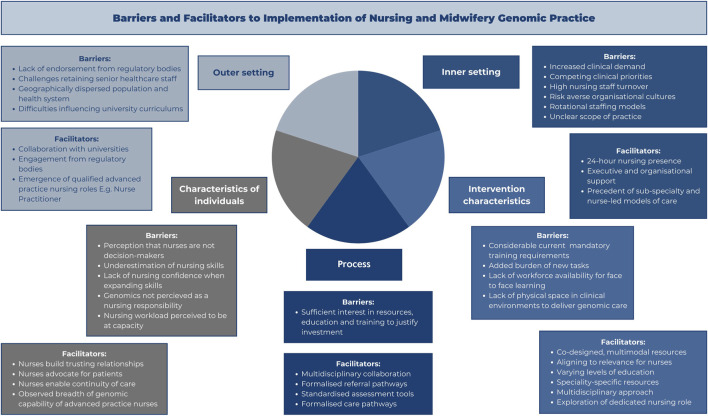

## Introduction

The uptake of genomic care into contemporary healthcare environments presents significant opportunities for the enhancement of diagnostic and treatment accuracy, thereby improving outcomes across various disease continuums (Bilkey et al., 2019). This signifies an important shift, whereby medical interventions can be more readily tailored to individual patients, facilitating more targeted treatments and enhancing patient care and personalised medicine (Masucci et al., 2024; Elzagallaai et al., 2023). While the utility of genomic care is established in fields such as oncology, reproductive health, and paediatric care, their integration into health systems faces several challenges.

In Australia, the number of genetic and genomic healthcare professionals, such as genetic counsellors and geneticists, is insufficient to meet current and future demand for these services (O'Shea et al., 2022; Nisselle et al., 2019). Upskilling the broader healthcare workforce to effectively identify appropriate patients, facilitate referrals, and interpret genomic data would alleviate the demand on specialist services, enabling them to focus on more complex cases. Without adequate investment, access to genomic services will remain limited, with lengthy wait times for Australians seeking access to these services (Rahman et al., 2022; Zebrowski et al., 2019). Additional concerns include data privacy, ethical considerations, and the absence of standardised protocols to ensure consistent and equitable access to genomic services (Vassy et al., 2015).

As genomic mainstreaming progresses throughout health services, patients increasingly expect their providers to have the knowledge and skills required to support them accessing appropriate services (Zoltick et al., 2024). In Australia, the National Health Genomics Policy Framework (2018–2021) identified the health workforce as key to incorporating genomic advancements within care (Australian Government, 2018). Despite being Australia’s largest healthcare workforce, research has shown that up to 75% of Australian nurses report significant gaps in their knowledge and confidence related to genomic testing, patient education, and effective communication about genomics (Alexander et al., 2024). This finding aligns with international literature, which identifies a global capability gap within the nursing healthcare workforce regarding genomic competencies (Gaff et al., 2017; McClaren et al., 2020). A key factor contributing to this gap is that the rapid advancements in genomic medicine and technologies have outpaced the education, training, and skill development of healthcare professionals (McClaren et al., 2020; Schluter, 2023). As a result, many nurses and other frontline providers feel ill-prepared to incorporate genomic information into patient care (Alexander et al., 2024; Schluter, 2023). This disparity poses a serious risk of underutilising the potential benefits of genomic care, leading to missed opportunities for early diagnosis, personalised treatment plans, and improved health outcomes (Jooma et al., 2019). This lack of skilled healthcare staff and established care pathways also risks perpetuating existing health inequities and limited access to genomic informed care (Khoury et al., 2022).

Effective integration of genomics into the Australian healthcare system requires a workforce with the knowledge, skills, and resources to deliver genomic care (Stark et al., 2019). Building such capacity may also enhance healthcare efficiency and sustainability by allowing specialised genomics services to focus on complex and emerging cases. Given that the nursing and midwifery workforce represents Australia’s largest and most accessible health professional group, they constitute a critical resource for the advancement and expansion of genomic care. Upskilling nurses and midwives could enable genomic care to be more readily embedded in routine clinical care and ensure broad and equitable access throughout Australia (Carpenter-Clawson et al., 2023; Limoges et al., 2024a). Therefore, this mixed-method study aims to clarify nursing and midwifery scope of practice in genomic care, identify barriers and facilitators to genomics mainstreaming, and identify the education and resource needs of nurses and midwives in Australia.

## Materials and methods

Our study consisted of a two-part, exploratory project, conducted across the state of Queensland, Australia (Hallingberg et al., 2018). Part 1 employed a state-wide workforce survey to establish the domains of practice for the Nursing and Midwifery workforce with respect to delivering genomic care. This survey was guided by a steering committee of experts in the areas of genomics, Nursing and Midwifery practice and research. Part 2 reflected on the findings of Part 1 through semi-structured interviews with key stakeholders, exploring the barriers and facilitators to the integration of genomic care into Nursing and Midwifery practice.

### Sample, recruitment and consent

Part 1 participants consisted of nurses and midwives as end-users of genomic practice. Participants were recruited through the professional networks of the steering committee and research team. The recruitment strategy involved using email invitations to >170 Nursing and Midwifery Directors for wider dissemination. The research team did not ask the Nursing and Midwifery Directors to respond with actions taken and therefore the precise survey response rate is unknown. However, to maximise response rates, a combination of purposive and snowball sampling techniques was utilised, with email invitations shared across all sixteen Hospital and Health Boards within Queensland, which employ approximately 40,000 nurses and midwives (Queensland Health, 2025). Study information was shared, and consent was obtained prior to participation in the survey. Part 2 employed semi-structured interviews with a range of key stakeholders, including medical staff, nursing managers, genetic specialists, multidisciplinary staff, and members of regulatory bodies and academic institutions. A sample range of 20–40 experts was targeted to reflect a broad cross-section of stakeholder perspectives (Malterud et al., 2016). Participants were recruited purposively via an email invitation and informed via word of mouth or recommendation from a previous participant. Study information and consent forms were provided to participants prior to participation. Consent for Parts 1 and 2 were guided by Chapter 2 of the National Statement on Ethical Conduct in Human Research 2023 (Nhmrc, 2023). Confidentiality was ensured throughout Parts 1 and 2 through data being de-identified and retained on a password protected hard drive. Interview consent was also verbally affirmed before and after the interviews were conducted.

### Data collection and analysis

The state-wide survey consisted of 31 domains of genomic practice, identified in previous research (Alexander et al., 2024). These domains encompassed genomic knowledge, understanding of testing and technologies, facilitation of testing, interpretation of testing, patient education and communication, supportive care, ethics, legal and social and professional development (Alexander et al., 2024). The term “genetic/genomic” was utilised interchangeably within the survey domains and the subsequent interviews, to enable broad and accessible discussions with a wide range of participants. Survey responses were categorised on a scale of “all, some, few or none” in relation to nursing and midwifery involvement in each genomic practice domain. Quantitative data were collected via REDCap (REDCap, 2025) software and exported into the Statistical Package for the Social Sciences (SPSS) Version 30 (Ibm, 2025). Descriptive statistics were generated to summarise key differences in demographic variables, using frequencies and percentages, rounded to the nearest whole number.

The semi-structured interviews were conducted to identify the barriers and facilitators to implementing the genomic practice domains discussed in the survey. Interview questions were guided by the expert steering committee and previous research conducted by the research team (Alexander et al., 2024). Full interview questions are reported in Supplementary File 1. Interviews were conducted and audio-recorded on the videoconference software Microsoft Teams (Microsoft Teams, 2023). Interviews ranged from 30 to 60 min in duration. Interviews continued until data saturation was reached, which was determined when no new themes or codes were found in additional interviews (Naeem et al., 2024). Interviews were transcribed verbatim and checked by the interviewer (MF) for completeness. Data were independently coded by two researchers (MF and BS) using both inductive and deductive approaches (Proudfoot, 2023). Data were coded manually by the researchers, without use of coding software. Discrepancies in coding were resolved by discussions with the lead researcher (KA), with consensus reached through joint review. Transcripts were analysed using qualitative content analysis (Hsieh and Shannon, 2005), with results organised per the Consolidated Framework for Implementation Research (CFIR) (CFIR, 2025; Damschroder et al., 2022). CFIR is a comprehensive framework that identifies and organises key factors that influence the effective implementation of interventions (CFIR, 2025). The outer setting of the CFIR includes the economic, political, and social context within which an organisation resides (Damschroder et al., 2009). The inner setting of the CFIR includes features of structural, political and cultural contexts within the organisation (Damschroder et al., 2009). The characteristics of individuals includes the cultural, organisational, professional and individual mindsets, norms, interests of those involved in the intervention, and their associated power and influence within the organisation (Damschroder et al., 2009). The characteristics of the intervention include both core and adaptable components related to the intervention and the organisation in which it is being implemented (Damschroder et al., 2009). The process domain of the CFIR involves a series of activities or strategies aimed at enabling the intervention (Damschroder et al., 2009).

## Results

### Survey

A total of eighty-one (n = 81) participants completed the survey. Respondents consisted of Registered Nurse or Midwife (22%), Clinical Nurse or Midwife (19%), Nurse Practitioner (10%), Clinical Manager (12%), Nursing Educator (10%), Nursing Researcher (6%), Nursing or Midwifery Director (16%) or Other (5%). Most (89%) participants were female, worked in a patient-facing role (63%) and had >20 years’ experience in healthcare (70%). Full survey participant demographics are presented in Table 1.

**TABLE 1:** Survey participant demographics.

Characteristic		Nurses and midwives	
		N (81)	%
Gender	Male	8	10
	Female	72	89
	Non-binary	0	0
	Prefer not to say	1	1
Years healthcare	<10	10	12
	10–20	14	17
	>20	57	70
Location of work	Metropolitan	57	70
	Regional	18	22
	Rural/Remote	6	8
Highest qualification	Certificate/Diploma	6	8
	Bachelor’s degree	27	33
	Post-graduate degree	48	59
Patient-facing role	No	30	37
	Yes	51	63
Currently using genetics/genomics in practice	No	67	83
	Yes	14	17
Primary role	Registered nurse/Midwife	18	22
	Clinical nurse/Midwife	15	19
	Nurse practitioner	8	10
	Clinical manager	10	12
	Educator	8	10
	Researcher	5	6
	Director	13	16
	Other	4	5
Area of practice	Oncology and haematology	20	25
	Maternity/Women’s health	11	14
	General medicine	8	10
	Other specialities^a^	42	51

^a^ Aged Care (n = 2), Cardiology (n = 2), Paediatric (n = 1), Clinical Service Design (n = 3), Education (n = 3), Emergency (n = 2), Endocrine (n = 1), Infectious Diseases (n = 2), Intensive care (n = 2), Nephrology (n = 1), Leadership/Management (n = 3), Respiratory (n = 3), Mental Health (n = 4), Metabolic (n = 1), Neurological (n = 2), Research (n = 2), Rural and Remote Health (n = 3), Safety and Quality (n = 2), Perioperative (n = 3).

### Domains of genomic-related care practice

**FIGURE 1:**
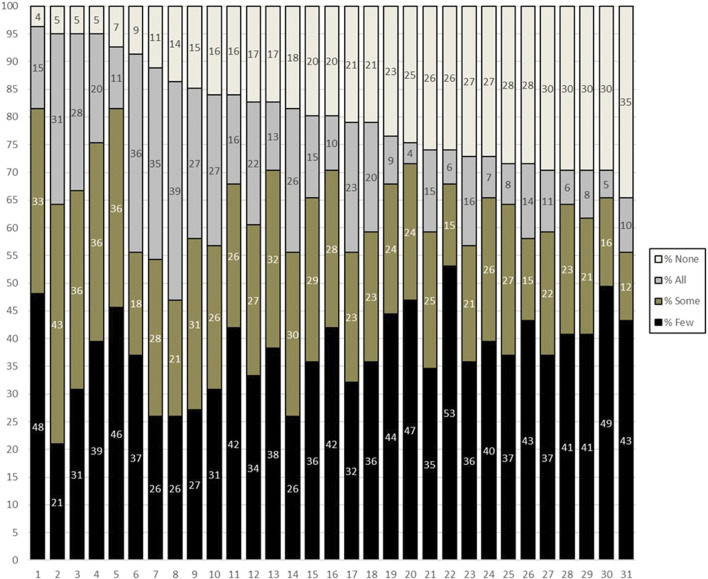
Survey results displaying level of nursing involvement in genomic-related care practice.

The domains of genomic practice identified in previous research (Alexander et al., 2024) are ranked and presented in Table 2. The ranked domains exhibited a clear progression, moving from awareness of genomic care (domains 1–8) to responsiveness to genomic needs (domains 9–18), and finally to proactive engagement in genomic care (domains 19–31). Notably, the proactive engagement activities emphasise where nursing and midwifery roles intersect with traditional genomic services. The survey found that the domains with the highest overall agreement included being able to identify clinical indicators of genetic susceptibility: 96% agreement (all; 15%, some; 33%, few 48%), be able to take a family history: 95% agreement (all; 31%, some; 43%, few 21%), have a general understanding of genomic information relevant to their practice area: 95% agreement (all; 28%, some; 36%, few 31%), and have a general understanding of genomic testing procedures and technologies relevant to their practice area: 95% agreement (all; 20%, some; 36%, few 40%), and being able to identify clinical indicators for genomic testing: 93% agreement (all; 11%, some; 36%, few 46%). The extent of nursing and midwifery involvement in each domain was situational, with most participants choosing “few” or “some” and lower proportions of “none” or “all”. The top three preferences of domains of practice for ‘all’ nurses and midwives to be involved in include assuring patients about the confidentiality of the information they provide about family history or their genomic information (39%) and recognising the ethical implications of genomic testing (36%) and advocating for patients’ access to desired genomic services (35%). The domain with the least agreement regarding nursing and midwifery involvement was being able to perform a pedigree analysis: 65% agreement (all; 10%, some; 12%, few 43%). Full survey results are reported in Figure 1.

**TABLE 2:** Domains of genomic practice.

Rank	Domains of practice
1	Identify clinical indicators of genetic susceptibility
2	Be able to take a family history
3	Have a general understanding of genetic/genomic information relevant to their practice area
4	Have a general understanding of genetic/genomic testing procedures and technologies relevant to their practice area
5	Identify clinical indicators for genomic testing
6	Recognise the ethical implications of genetic/genomic testing
7	Advocate for patients’ access to desired genetic/genomic services
8	Assure patients about the confidentiality of the information they provide about family history or their genetic/genomic information
9	Consult other members of the multidisciplinary team about genetic/genomic-related issues
10	Access current and reliable genomic/genomic information
11	Identify gaps in knowledge about genetics/genomics
12	Participate in genetic/genomic-related professional development activities
13	Decide which patients would benefit from a genetic/genomic service referral
14	Respond appropriately to patients who experience distress related to genetic/genomic issues
15	Provide information to patients about genetic/genomic testing
16	Answer questions about genetics/genomics by a patient or their family
17	Consult with a nursing or midwifery peer about genetic/genomic-related issues
18	Incorporate genetic/genomic information into care provided
19	Educate a patient or their family about genetic susceptibility and genetic/genomic testing
20	Provide genetic/genomic education and mentoring to peers
21	Facilitate a genetic/genomic service referral
22	Interpret genetic/genomic testing results
23	Participate in the conduct of genetic/genomic-related research
24	Provide information on the risks, benefits, and limitations of genetic/genomic testing
25	Guide patients on genetic/genomic-related decision making
26	Obtain patient consent for genetic/genomic testing
27	Contact genetic/genomic services for a consultation for a patient
28	Identify new developments in genetics/genomics
29	Obtain patient consent for genetic/genomic testing for research
30	Communicate the results of genetic/genomic testing to a patient
31	Be able to perform a pedigree analysis

### Semi-structured interviews

There were thirty-two (n = 32) participants who individually participated in an in-depth semi-structured interview. Each interview ranged from 30 to 50 min in duration. Participants included Executive Director Nursing and Midwifery (n = 10), Medical (n = 1) and Allied Health (n = 1); Genetics specialist (n = 5); Oncologist (n = 1); Member of a professional organisation or tertiary education institution (n = 6); Nurse Unit Manager (n = 2); Clinical Nurse Consultant (n = 2); Nurse Practitioner (n = 2) and Registered Nurse (n = 1). Participants represented a range of speciality areas, including executive leadership, Aboriginal and Torres Strait Islander health, genetics, education, regulatory bodies, respiratory, cardiology, internal medicine, paediatrics, mothers and newborns, oncology, and haematology. Full participant characteristic presented in Table 3.

**TABLE 3:** Interview participant characteristics.

Profession	n (%)
Nursing and midwifery	18 (56%)
Medical	2 (6%)
Allied health	1 (3%)
Professional organisation and/or tertiary education	6 (19%)
Institutions	5 (16%)
Genetics practitioners	32 (100%)
Total	​
Speciality area	
Executive (nursing and midwifery)	5 (16%)
Executive (heart and lung)	2 (6%)
Executive (aboriginal and torres strait islander health)	2 (6%)
Executive (education)	1 (3%)
Medical director (internal medicine)	1 (3%)
Executive (allied health)	1 (3%)
Genetics specialist (geneticist or genetic counsellor)	5 (16%)
Oncologist	1 (3%)
Educator (nursing)	1 (3%)
Professional organisation and/or tertiary education	6 (19%)
Institutions	2 (6%)
Nurse unit manager	2 (6%)
Clinical nurse consultant	2 (6%)
Nurse practitioner	1 (3%)
Registered nurse	32 (100%)
Total	​
Sex	
Female	24 (75%)
Male	8 (25%)
Total	32 (100%)

### Outer setting

Factors facilitating the integration of genomic care from the outer setting included collaboration with university programs, notably the incorporation of genomic education into undergraduate (pre-registration) nursing curricula, alongside the availability of additional postgraduate training opportunities. The expanded role and growing numbers of Advanced Practice Nurses, such as Nurse Practitioners, were also identified as potential key facilitators of genomic referrals. Furthermore, engagement and collaboration with regulatory bodies were recognised by some participants as important drivers of workforce practice change. Conversely, regulatory bodies and organisational structures were viewed by some participants as barriers to practice change, because of a lack of endorsement for nursing roles in genomic practice. Additional barriers included challenges retaining senior staff, high staff turnover, a geographically dispersed population and difficulties influencing university curriculums.

One participant discussed the need to further integrate general genomic education into university curriculums:

“We really do need to start at the undergraduate level… there’s a little bit of genomics in the current undergraduate curriculums, but… maybe the universities we should have some discussions about building upon that”.

(Nursing Director, Education).

Another participant reflected on the role of both professional bodies and tertiary institutions in supporting front line cancer clinicians, such as nurses and doctors:

“We need to support those clinicians… who are in that space so that they can have the knowledge and skills… they’re protected in terms of you know, the medical, legal and regulatory environments”.

(Nursing, Professional Organisation).

### Inner setting

Facilitators identified from the inner setting included the role of nurses as a 24-h presence within the hospital, having access to appropriate information and resources, having executive and organisational support for practice changes, and the acknowledgement that they were numerous examples of genomic practice currently within the service. Barriers identified within the inner setting include increased clinical demand with competing priorities which increases cognitive and physical workloads, a risk averse organisational culture, reliance on rotational staffing models, increased nursing staff turnover, and a lack of clarity regarding nursing scope in the field of genomics.

One participant discussed the role of nurses as a continuous presence for patients:

“They’re the 24-h presence, everybody else is relatively episodic in their care… so nurses have this really sort of coordinating role I feel, which is important, but under recognised I think”.

(Nursing, Executive Director).

Another participant highlighted the constraints imposed by system-level factors on clinician-led change:

“Things at the system level, you know you’ve got this really enthusiastic clinicians, which will do this wonderful job in their sphere of influence… but change cannot run by enthusiasm alone”.

(Nursing, Executive Director).

### Characteristics of individuals

A range of individual characteristics were associated with facilitating genomic practice, with particular emphasis on the nursing role. Nurses were recognised for developing trusting relationships with patients, their role in patient advocacy and ensuring continuity of care. Participants acknowledged that nursing roles, along with their associated skills and knowledge, are evolving. The expanded scope of Nurse Practitioners and current nursing involvement in consent processes outside of genomic care were also identified as facilitators. Several individual characteristics were identified across all participant groups as barriers to genomic practice, including the perception that nurses are not decision-makers, the underestimation of nursing skills, and a lack of confidence among nurses. Additionally, a lack of clarity regarding the nursing scope of practice was acknowledged, which may lead some nurses to view genomics as outside of their responsibilities.

One participant reflected on the influence of historical perceptions of nurses’ roles and abilities:

“Nurses were not considered [historically] to be autonomous decision makers … there are still components of that”.

(Executive Director, Nursing).

The importance of the nursing role in building trusting relationships with patients was identified by several participants as a facilitator:

“[Nurses] have those relationships and that trust; people will come and see you”.

(Nursing Director, Cardiology).

### Intervention characteristics

Facilitators to genomic care identified within intervention characteristics included co-designed, multimodal education and clinician resources; aligning the relevance of education and training with individual roles; and offering education programs with varying levels of detail. Participants emphasised the importance of accessible, online training courses that cater to different levels of expertise. Participants frequently expressed that genomic knowledge, and skills should be speciality-dependent, with tailored resources. A multidisciplinary approach was consistently viewed as best practice, with widespread support for expanding Advanced Practice Nursing roles, such as the Nurse Practitioner role to facilitate genomic care delivery and provide nursing leadership. Barriers related to intervention characteristics included competing demands on clinicians’ time between mandatory and non-mandatory training, the added burden of new tasks on already high workloads, lack of workforce availability for face to face training, and lack of physical space within the clinical environment to deliver genomic care.

Several executive-level staff emphasised the importance of involving nurses and midwives in the co-design and development of new roles and responsibilities to ensure their engagement and support throughout the implementation process:

“There’s significant competing priorities for nurses and midwifes… [they are] called on to do more and more… so I think that genomics is going to be perceived as something that either doesn't sit within their roles or is less important than other things”.

(Executive Director, Nursing).

This was supported by a second participant who reflected that a dedicated Nurse Practitioner or Clinical Nurse Consultant would be beneficial in meeting future demands for genomic testing.

“I really think having someone built into the clinic like one of these nurse practitioners or someone able to take on that role would be really helpful because that would be a huge volume of people who would meet testing criteria”.

(Educator, Regulatory body).

### Process

Facilitators of genomic care within the process domain included genuine multidisciplinary collaboration, the implementation of formalised referral pathways, and the use of standardised assessment tools to support consistent practice. To support nurses in delivering genomic-informed care, participants reported that access to guidelines or checklists would be beneficial. These processes were seen to streamline care delivery and enhance communication across teams. In contrast, a key barrier identified was the potential lack of sufficient demand or interest to justify the provision of education and training, which may limit learning opportunities for nurses and midwives in genomic care.

One participant reflected on the benefit of having genomic tasks integrated into everyday nursing practices:

“Whether things can be integrated to be a part of their everyday tools and assessment processes”.

(Nursing Director, Cardiology).

Another participant discussed the low enrolment numbers as a barrier to delivering education and training:

“There’s almost a commercial aspect to it, I can't run a course with less than five people or six people in it because … It's not worth the investment of the educators to be able to deliver that training or education”.

(Educator, Professional Organisation).

## Discussion

To our knowledge, this study is among the first to explore the barriers and facilitators impacting nursing and midwifery engagement in genomic care delivery in Australia. Our findings indicate broad agreement that nurses and midwives should participate in genomic healthcare delivery, to varying extents. Survey responses indicate that nurses and midwives should have some capability in 18 of the 31 genomic-related practice domains previously identified (Alexander et al., 2024). However, several challenges to the integration of genomic care into nursing and midwifery practice were highlighted, including unclear scope and responsibilities, organisational and structural barriers, and the need for tiered educational resources and implementation pathways. Key facilitators to support this integration included the establishment of dedicated nursing or midwifery roles, tailored educational programs, co-design throughout all phases of the model of care development, and active collaboration with universities and regulatory bodies. Key themes identified included role clarity, scope definition and integration into current practice, and the necessary education and resourcing.

### Role clarity, scope definition and integration into practice

Role clarity, scope of practice, and the practicalities of integration into practice were consistently discussed by participants. Overall, participants highlighted the need for clear role definitions and a well-defined scope of practice pertaining to genomic related healthcare to successfully integrate genomic care into nursing and midwifery practice. There was an overwhelming desire for the establishment of a dedicated role to support nurses in genomic-related healthcare. This role was characterised as having a clearly defined scope of practice and should be supported by the development of appropriate resources, clinical pathways, and training programs. These findings are consistent with existing literature that suggests without role clarity, there are risks of role confusion, fragmented care, and missed opportunities to harness the full potential of genomics in improving patient outcomes (Thomas et al., 2023; Gusen et al., 2025). Furthermore, when nurses and midwives are provided with explicit role expectations and the necessary resources, they are better equipped to integrate new skills and technologies into their current roles (Brault et al., 2014; Lankshear et al., 2016). The genomic care domains demonstrated a clear progression, from foundational awareness of genomic care to responsiveness to patients’ genomic needs, and ultimately to proactive engagement in genomic practice. This aligns with international literature, which indicates that general nurses and midwives primarily engage in the early awareness and responsiveness stages, while specialist nurses and genetic counsellors operate at the proactive engagement level (Skirton et al., 2010). Consistent with this, survey findings showed the lowest agreement for nursing and midwifery involvement in domains requiring advanced technical skills, such as pedigree analysis, consent processes, and referrals. While there was broad support for nurses taking family histories, there was notably less agreement regarding their role in performing pedigree analysis. This discrepancy reflects a debate among health professionals, with some asserting that comprehensive family histories necessitate pedigree analysis, while others contend it is not essential (RACGP, 2025; Bylstra et al., 2021). Clarifying such distinctions in genomic care delivery will be crucial for defining the appropriate scope of nursing and midwifery practice and understanding how these roles intersect with genetic specialist services.

International evidence from the United Kingdom and United States reports that nurses and midwives are recognised as integral to the mainstreaming of genomic care and are expected to routinely perform advanced practice domains (Carpenter-Clawson et al., 2023). In these contexts, expanding the scope of genomic practice of frontline clinicians, such as nurses and midwives, beyond specialist-only delivery has been identified as essential for effectively embedding genomics into routine care (Carpenter-Clawson et al., 2023). To facilitate this, nurses and midwives in the United Kingdom have developed competencies in genomic education, risk assessment, referral pathways, family history collection, and consent processes, highlighting their evolving role in genomic healthcare (Carpenter-Clawson et al., 2023; Shepherd et al., 2014). These international variations in genomic care delivery highlight the importance of establishing role clarity and scope definition for nurses and midwives as Australian health services mainstream genomics. Clarifying these roles is essential to enhance clinician confidence, enable effective patient education and strengthen multidisciplinary collaboration (Gusen et al., 2025; Brault et al., 2014).

### Knowledge, education, and training

The second set of themes identified was the need to adequately build knowledge and provide education and training. The findings highlight the importance of co-designing resources to ensure they are multi-level, accessible to all clinicians, and accommodate varying levels of informational depth and learning styles. Participants emphasised that knowledge and skills related to genomics are speciality-specific, with advanced practice nurses and midwives, such as nurse practitioners, requiring more in depth or formalised education versus frontline nurses such as registered and clinical nurses. To address this, participants suggested a tiered or stepped educational approach, whereby brief, online modules would provide foundational knowledge for general clinicians, while more formalised training and competency-based education would be reserved for advanced practice roles. This approach aligns with existing literature, which highlights the benefits of differentiated learning pathways in enhancing knowledge retention and skill development by aligning educational content with clinicians’ roles and responsibilities (McLaughlin et al., 2024). Several participants identified a gap in pre-registration nursing curricula, noting the current absence of genomics content (Alexander et al., 2024). The survey results indicated the highest levels of agreement that all nurses should possess competencies related to assuring patients of confidentiality, recognising the ethical implications of genomic testing, and advocating for patient access to genomics. This suggests that integrating these conceptual dimensions into pre-registration education may be more beneficial than universities teaching advanced genomic skills in the pre-registration context. Furthermore, the interviews highlighted the need to clearly define the scope of genomics education and training appropriate in the pre- and post-registration settings. The findings indicate that nurses and midwives require both enhanced genomic knowledge and practical competencies to ensure the safe delivery of genomic care, necessitating coordinated collaboration between academic institutions and regulatory bodies (Dheensa et al., 2017; Sahan et al., 2024).

In response to the identified education and training needs of clinicians, the co-design and coproduction of educational content in partnership with key stakeholders, such as consumers, geneticists, and genetic counsellors in this context, has been shown to improve the relevance and applicability of training (Peddle et al., 2025; Brand et al., 2023). Collaborative design processes have also been shown to more closely align with patient needs and support multidisciplinary collaboration (Peddle et al., 2025). Importantly, this model of education supports ongoing professional development and facilitates progression from basic genomic knowledge to advanced clinical application, as necessary (Brand et al., 2023; O'Connor et al., 2021). This educational approach responds to the current capability gap in the nursing and midwifery workforce, by providing resources ranging from general knowledge to advanced practice, promoting more effective integration of genomics into routine healthcare delivery (Nisselle et al., 2024; Limoges et al., 2024b).

### Systemic, organisational and resource support

A significant factor impacting the integration of genomic care within nursing and midwifery roles was identified as health system, organisational, and resource support. Participants highlighted critical system-level factors, including the need for enhanced infrastructure and targeted workforce investment to enable the expansion of clinicians’ roles. Additionally, support and active engagement from regulatory bodies were recognised as essential facilitators for effective workforce transformation. This aligns with the wider literature, which demonstrates that active engagement and endorsement from these bodies is necessary to establish professional standards, scope of practice guidelines, and credentialing frameworks that legitimise and sustain new genomic roles within nursing and midwifery (Burns et al., 2019; Kirk et al., 2014). Without such regulatory support, workforce change risks being inconsistent and lacking formal recognition, thereby limiting its impact (Chiu et al., 2024). Similarly, engagement with key genomics service providers was identified as necessary for a coordinated approach (Basnayake Ralalage et al., 2024). Adequate resource support was also recognised as a critical factor in ensuring equitable access to genomic care across populations. Consistent with the wider literature, participants emphasised that establishing dedicated nursing and midwifery roles focused on genomics would likely increase the uptake of genomic services and expand the number of patients engaging with genomics-informed clinicians (Limoges et al., 2024a). This is largely attributed to the consistent presence of nurses and midwives within healthcare settings, as well as their role as often the initial point of contact for patients accessing health services (Limoges et al., 2024a; Fasaye et al., 2021). These findings underscore the critical need for a coordinated, multi-level approach to mainstreaming genomics, which actively involves key stakeholders across the health system, organisations, and regulatory bodies. Such collaboration is essential to ensure the equitable and effective integration of genomic care within nursing and midwifery practice.

### Implications for clinical services

Findings from this study have important implications for health services in Australia and internationally. In the United Kingdom and the United States, nurses and midwives play a key role in advancing the mainstreaming of genomic care (Carpenter-Clawson et al., 2023). To align with these models, Australian health services must recognise the critical role of this workforce. Firstly, failing to adequately enable nurses and midwives in genomic care poses significant risks, including missed opportunities for early identification of genomic needs, patient education, and personalised interventions that could improve health outcomes.

Without the involvement of nurses and midwives, clinical services risk becoming overwhelmed, as specialist-led models alone are unlikely to meet the growing demand for genomic testing and interpretation across Australia (Nisselle et al., 2021). Importantly, the upskilling of nurses and midwives in genomic care presents substantial opportunities to expand access to genomic care and enhance multidisciplinary collaboration (Limoges et al., 2024a). The transition to mainstreaming genomic services represents a significant shift in clinical services, necessitating that nurses and midwives play an active role in the design, implementation, and delivery of new genomic-based interventions (Carpenter-Clawson et al., 2023; Berkman et al., 2025). Rather than simply absorbing emerging genomic responsibilities, nurses and midwives require dedicated education, training, and resources to ensure safe and effective practice (Alexander et al., 2024). To fully realise the benefits of genomic care, this study, along with the broader literature supports the use of tailored, co-designed workforce development programs, delivered through differentiated educational and training pathways that address the diverse scope and competency needs of clinicians (Shepherd et al., 2014; Brand et al., 2023).

This study has several limitations to consider. Although the survey was widely disseminated to >170 Nursing and Midwifery Directors across Queensland, it received only 81 responses. This relatively low response rate may reflect a broader lack of engagement with genomic care within the nursing and midwifery workforce. Despite this, the demographic characteristics of both the survey and the interview participants appear broadly representative of Australian nursing and midwifery professions. Additionally, the survey instrument and interview questions were not based on previously validated questionnaires, reflecting the exploratory in nature of this study.

## Conclusion

The integration of genomics into Australian health services presents substantial opportunities to improve diagnosis and treatments but relies on an upskilled workforce capable of delivering genomic-informed care. This study identified support for nursing and midwifery involvement in 18 of 31 genomic practice domains, revealing the need for all nurses and midwives to have general genomic awareness, with some expected to be responsive to genomic needs and some to provide proactive genomic care. Fewer nurses and midwives were expected to engage in highly technical genomic activities. Several barriers to integration were noted, including unclear roles, systemic and organisational challenges, and insufficient education and resources. Key facilitators included dedicated nursing and midwifery roles, co-designed education and workforce development programs, and strong stakeholder collaboration. These findings underscore the need for clearly defined roles and scopes of practice, supported by tailored, co-designed workforce development initiatives and differentiated learning pathways. Such strategies are crucial for Australian health services to fully realise the benefits of accessible genomic care.

## Data Availability

The raw data supporting the conclusions of this article will be made available by the authors, without undue reservation.
