# Supplementary material for: Role of nursing and midwifery in mainstreaming genomics in Australia: mixed-methods study exploring scope of practice and strategies for implementation
Source: Front Genet. 2026 Jan 5;16:1717520. doi: 10.3389/fgene.2025.1717520 (PMC12812403; doi:10.3389/fgene.2025.1717520)
Supplement: Supplementary file 1 [file Supplementaryfile1.docx]

GINAM: Interview Guide

You’ve signed the consent form however, I wanted to remind you that you will be audio recorded during the interview and everything you say will remain confidential and if you want to withdraw your participation at any time you are welcome to do so. Is it okay to proceed and do you have any questions before we begin?

Wonderful – so the purpose of this project is to first establish key domains of practice for nurses and midwifes with respect to genetics and genomics and secondly to inform the development of an implementation toolkit to help individual service lines appropriately integrate genetic/genomic care into their services*.*

I will just share my screen now and put the domains of practice on the screen for you to refer to and the key topics of discussion for today.

To start with

1. Can you tell me a little bit about your experience in healthcare? We won’t use this information in publications but may help us understand the context of your answers.

*(PROMPTS: how long have you been in it, what is their position, service line experience, use of genomics)*

1. What barriers do you anticipate we may encounter when implementing any of these domains into practice for nurses and midwifes?

(PROMPT: What service line, people, resources/infrastructure, health systems, policies, funding, staffing, any particular domains trickier than others)

2a. How might this differ for different services?

1. What do you think would enable a smooth implementation?

(PROMPT: provide your biggest tips, how would you go about this, what service line, what resources, what changes to infrastructure, funding, redesigning workflows, gap analysis, who decides on scope of practice)

3a. How might this differ for different services?

1. So based on this then where would you start?
2. Refer to answers can you explain why you said no to XYZ

*(PROMPTS: are there any deal breakers, any you are concerned about, any you would add in)*

1. Would we benefit from speaking to anyone else in your network who might be in a position to influence or provide insight on the implementation of nursing and midwifery genomic capabilities that we should talk to?
2. Anything else you want to tell us?
